# BIR: Biomedical Information Retrieval System for Cancer Treatment in Electronic Health Record Using Transformers

**DOI:** 10.3390/s23239355

**Published:** 2023-11-23

**Authors:** Pir Noman Ahmad, Yuanchao Liu, Khalid Khan, Tao Jiang, Umama Burhan

**Affiliations:** 1 School of Computer Science and Technology, Harbin Institute of Technology, Harbin 150001, China; 2 Department of Computing Science and Mathematics, University of Stirling, Stirling FK9 4LA, UK

**Keywords:** electronic health record, biomedical information retrieval, transformers, healthcare, cancer treatment

## Abstract

The rapid growth of electronic health records (EHRs) has led to unprecedented biomedical data. Clinician access to the latest patient information can improve the quality of healthcare. However, clinicians have difficulty finding information quickly and easily due to the sheer data mining volume. Biomedical information retrieval (BIR) systems can help clinicians find the information required by automatically searching EHRs and returning relevant results. However, traditional BIR systems cannot understand the complex relationships between EHR entities. Transformers are a new type of neural network that is very effective for natural language processing (NLP) tasks. As a result, transformers are well suited for tasks such as machine translation and text summarization. In this paper, we propose a new BIR system for EHRs that uses transformers for predicting cancer treatment from EHR. Our system can understand the complex relationships between the different entities in an EHR, which allows it to return more relevant results to clinicians. We evaluated our system on a dataset of EHRs and found that it outperformed state-of-the-art BIR systems on various tasks, including medical question answering and information extraction. Our results show that Transformers are a promising approach for BIR in EHRs, reaching an accuracy and an F1-score of 86.46%, and 0.8157, respectively. We believe that our system can help clinicians find the information they need more quickly and easily, leading to improved patient care.

## 1. Introduction

Biomedical research can be catalyzed by the vast amount of clinical data contained in electronic health records (EHRs). Although EHRs provide many benefits, leveraging them for cancer research remains challenging [1]. Since many clinical details (up to 80% by some estimates) are captured in free-text notes, converting them into a computable form is difficult [2]. National health reform initiatives have aimed to improve coordination and communication between care sites. The discharge communication from the hospital plays an important role here, since it informs the development of the care plan in the next care setting. Despite this, providers report that poor discharge communication leads to a lack of communication between providers, medication discrepancies, and avoidable 30-day readmissions. The content and format of discharge communications vary substantially across institutions due to the limited standards that inform their creation [3,4]. Furthermore, the EHRs’ advent and spread have further increased this inconsistency. The lack of a consistent structure results in most discharge communications being composed of mainly free-text or “unstructured” data.

In addition, unstructured data that are documented without standard content qualifications are often recorded as free text [5]. A structured dataset, on the other hand, is usually entered into discrete data fields with established standards for responses, parameters, or conditions (e.g., age, weight). Despite providers’ reliance on unstructured data when communicating plan-of-care components within discharge communications, quality assessors or researchers have trouble finding those components with reliability. Unstructured communication components are difficult to measure reliably to determine baseline status. Quality measurement is hindered by the issue of unstructured data, according to Healthcare Research and Quality. A treatment-administered medical problem relationship exists between clinical text, Lexix, and congestive heart failure, as shown in Table 1.

In the following sample, $S_{1}$, $S_{2}$, $S_{3}$, and $S_{4}$ present a variety of diseases and their relationships based on biomedical or clinical domains. $S_{1}$ examples include Lexix and congestive heart failure, $S_{2}$ breast cancer (http://www.who.int/cancer/en/, accessed on 6 January 2023) [6] with COVID-19, $S_{3}$ lungs with a weak immune system, and $S_{4}$ pneumonia. Several rural areas are experiencing serious shortages of doctors due to a lack of doctors in those areas [7]. The development of natural language processing (NLP) tools has been devoted to helping researchers use free text within EHRs [8]. Therefore, NLP remains promising for oncology research, but its well-known use is still limited. However, the quality of NLP results is mixed, with some conceding the intricacy and “inherent difficulty of natural language processing in this domain”. Furthermore, to understand temporal relationships, ambiguous abbreviations, and anaphoric references, this complexity is a result of a variety of factors [9,10]. Therefore, these systems perform best when tailored to specific tasks and domains, so large manual annotation datasets are needed for new use cases. Additionally, limiting NLP systems is a lack of available experts. All U.S. hospitals have used EHRs since 2015 as the official standard for clinical records [11,12]. However, to increase an American hospital’s efficiency in processing and using patient information, there is a need to research new technologies for medical text. Thus, by allowing patients to quickly access this digitized information, diagnoses could be made more accurately and therapeutic treatments assigned [13]. Nevertheless, most of the present studies on search are dedicated to the World Wide Web (WWW, Web 2.0) and individual resources, and they cannot be openly useful to search over big data (big clinical text). However, Web searching over patient records agrees with leveraging the context to recover search significance. The clinical field and its leading concepts describe this context. It can be signified as a field/conception index and leveraged to enable further innovative essential text search [14]. In fact, numerous current sites have highlighted structural search over text as an area of rising awareness to the data mining community [15].

In this study, we aimed to evaluate BIR in EHR; the patient or doctor, in predicting treatment, could reprocess the existing information of symptoms in the medical records, associated health analysis, and medical diagnosis. Clinical texts contain incomplete or fragmented sentences, making extracting relations and retrieval entities harder. Due to manual feature engineering, hundreds of features are used in these SOTA methods [16]. We outperform the current models using a fraction of features. Nevertheless, the feature is used in our model, which is straightforward to reproduce and adjust to data sources. A state-of-the-art classification-based approach is also investigated over n-gram features, rich features, and their combination as a way to handle the BIR problem of the datasets.

This study proposed contributions as follows:

- We propose novel techniques for biomedical information retrieval of related or similar EHR between medical problems during symptom detection in existing information through testing and predicting the treatment in hospital clinical procedures.
- The proposed approach was evaluated on a dataset of EHRs and found to be able to outperform state-of-the-art BIR systems on a variety of tasks, including medical question answering and information extraction. The proposed approach is able to learn the semantic relationships between words in biomedical documents, which is essential for effective BIR.
- We evaluated our mechanism on a dataset of clinical texts and found that it was able to outperform state-of-the-art attention mechanisms on a variety of tasks, including medical question answering and information extraction.
- Clinical texts are often long and complex, with a lot of medical jargon. This makes it difficult for traditional attention mechanisms to focus on the relevant parts of a sentence. We evaluated our mechanism on an Integrating Biology and the Bedside (I2B2) dataset of clinical texts and found that it was able to outperform state-of-the-art attention mechanisms on a variety of tasks, including medical question answering and information extraction.

The rest of the paper is organized as follows: In the following section, we discuss related work. In Section 3, we discuss our proposed model and its implementation. Moreover, at the end of Section 3, we provide the details of the linear segment attention layer (Section 3.3.2). Section 4 proposes the best performance results and compares them with various other models. Finally, Section 5 comprehends our study’s conclusion, and also highlights the future research direction.

## 2. Related Work

IR is the process of finding and retrieving information from a collection of documents. However, BIR is a domain-specific IR application that is considerably dissimilar from other domains, specifically related to the biomedical domain [17]. Moreover, BIR models are developed over almost 60 years, developing from Boolean, vectorial, probabilistic, language, and learning-to-rank (LTR) models, to further neural models. Therefore, traditional BIR models rely on a lexical method based on bag-of-words (BoW), but this method suffers from semantic gap and vocabulary mismatch problems. Semantic search is a more recent approach to IR that addresses these issues by improving query and document illustrations to increase their level of understandability and acting a new meaningful document query identically driven by semantics. However, semantic search is based on a combination of structured knowledge resources (e.g., thesaurus, ontologies, and knowledge graphs) and unstructured data in the form of raw textual corpora.

### 2.1. Artificial Intelligence (AI)–Assisted Tools

An AI-assisted tool provides clinicians with a centralized resource for identifying, summarizing, and contextualizing pertinent research studies. ML techniques, such as Quertle and Meta, have been used in medical proof searches, but do not betray their intended purpose of extracting precise information from citations [18]. Therefore, this task is accomplished with an arrangement of focused text mining, NLP, natural language understanding (NLU), and ML to extract, filter, and rank information from reliable sources.

Furthermore, NLP/NLU and ML have focused on features of our system, comprising ML to categorize abstracts, medication–attribute connection in clinical narratives, identification of clinical research evidence, or PubMed-wide annotations [19,20,21]. Saiz et al. [22] designed Watson Oncology Literature Insights (WOLI) to support clinicians in the training of evidence-based medicine (EBM) by classifying related and appropriate research information in clinical oncology and peer-reviewed literature. Moreover, clinical information can be contextualized using WOLI using a particular patient situation or cohort to provide clinicians with directed information. In spite of this, the system circumvents the problem of signal-to-noise that arises from manual operations. Therefore, this study describes the system architecture and presents an evaluation of its performance.

Furthermore, to bridge that gap, this study established an AI-assisted method to automate deep learning analysis in oncology. The system is capable of ranking and purifying BIR for a specific clinical situation in oncology, mining and succeeding the related clinical results, and corresponding to most of the related articles, to a set of patient features.

### 2.2. Word-Level Attention Mechanisms

Attention mechanisms have been broadly used in NLP linguistic resources and tools, which utilize word-level attention mechanisms [23]. An NLP model dynamically adjusts each word’s weight based on the text’s content features, together with long short-term memory (LSTM) units, achieving results to the state of the art. Lin [24] proposed an attention-based model with a convolutional neural network (CNN) for distant supervised RE in sentence-level input data. 

During human diagnostics, the hypothesis formulation and evidence gathering phases are involved [25]. In some cases, patients complain of symptoms first; then the doctor performs some tests on the patient, and finally, he makes the judgment and offers a cancer treatment based on the test results and his medical knowledge [26,27]. Furthermore, MYCIN, a system for supporting early medical diagnosis, is based on human diagnostic procedures. However, MYCIN is a rule-based expert system for diagnosing diseases. Moreover, in the use of the system, the user (clinicians) must input the symptoms of the patient. Thus, the system will infer the diseases of the patient according to the symptoms and the rules built into the system. However, it is expensive to develop this type of system since the domain expert and the knowledge engineer must work closely together. Additionally, this system narrows down the specific diseases identified. Currently, diagnosis support systems are developing rules based on medical data in EHRs. Analyzing medical records and medical images for extracting rules or relations has been performed using techniques such as data mining, fuzzy sets, and rough sets [28]. As a result, these types of systems are considered cheaper than previous types of expert systems. Additionally, these systems can be kept current through the use of current hospital data and knowledge.

### 2.3. Pretrained Language Models (PLMs) for Summarization

Pretrained language models (PLMs) for text summarization in the general domain are a well-researched area, with many efficient methods, such as BERTSum [29]. However, El-Kassas et al. [30] offered a fine-tuned BERT encoder and a GPT-2 decoder for both the extractive and abstractive summarization of the COVID-19 literature. Furthermore, Du et al. [31] suggested BioBERTSum, which used domain-aware PLMs as the encoder and fine-tuned it on the biomedical extractive summarization task. In a study, Aaditya et al. [32] evaluated BERT’s performance on MIMIC-III discharge notes labeled with the International Classification of Diseases (ICD-9) for the extraction of extractive summaries from electronic health records. Moradi et al. [33] grouped contextual embeddings based on the BERT encoder into groups and selected the most informative sentences to generate the final summary of an unsupervised extractive summary in the biomedical domain. In addition, Padmakumar et al. [34] recommended an unsupervised extractive summarization model, which used the GPT-2 model to encode sentences and pointwise mutual information to analyze the semantic likeness between sentences and documents.

Text summarization based on PLMs is one of the most well-researched areas of computer science today, in which many efficient methods have been proposed. Existing research includes BERTSum [29], BioBERTSum [35], LABSE (language-agnostic BERT sentence embedding) [36], and ICD-9 MIMIC-III discharge notes [37] decoder in the biomedical domain. These domains have aimed at encoding and fine-tuning the input documents so that extractive summarizing could be extracted. In addition to GPT-3, Reformer [38], and DistilBERT [39], several other recent architectures have efficient language modeling, reliability, and performance metrics. BERT encoder and contextual embeddings of sentences were grouped using hierarchical clustering algorithms. They were then selected as the most informative sentences from each group to become the final summary for the biomedical domain [40]. In clinical NER, thus, Qiu et al. [41] stated that the ultimate goal is to identify and classify clinical terms such as symptoms, an exam, a cancer treatment, or disease in other words; their objective is to recognize and classify them. There is additional literature available about biomedical information retrieval techniques, and limitations are listed in Table 2.

In addition, DL algorithms focused on apprehending transitions concerning hidden states, such as bidirectional long short-term memories (BiLSTMs), recurrent neural networks (RNNs), and conditional random fields (CRFs). Information extraction and retrieval tasks are succeeded using pretrained transformer models, with BERT as one prominent example [51]. Therefore, DL algorithms using neural network models are used to solve IR, RE, and other data mining problems and simulate entity relationships. IR and IE tasks use components such as attention and BiLSTMs [17,52]. Similarly, SciBERT [53], BioBERT [54], and ClinicalBERT [55] have been modified and retrained on explicit domains, such as the biomedical domain, to improve domain specificity further. RoBERTa is used in particular NLP contexts, which have increased in healthcare mining, such as identifying bacteria–biotope relations, predicting hospital readmission, and normalizing biomedical information [56,57]. The ability to summarize biomedical text information is one of the most important duties for a reader to be able to comprehend an ever-growing amount of biomedical information.

This study proposes a two-part model that combines information retrieval from EHR and medical websites (such as https://www.medscape.com/ and https://www.smartpatients.com/, accessed on 18 February 2023) with the use of state-of-the-art language models (i.e., RoBERTa and BioBERT) trained on biomedical text corpora. Therefore, the model is able to achieve strong results on various NLP tasks, such as named entity recognition, coreference resolution, and semantic similarity. However, the study is limited to the English language and does not consider multilingual architectures. Moreover, future work could extend the model to other languages and could also consider multilingual architectures in order to enable inclusive e-health and improve patient participation in healthcare.

## 3. Method

**Figure 1:**
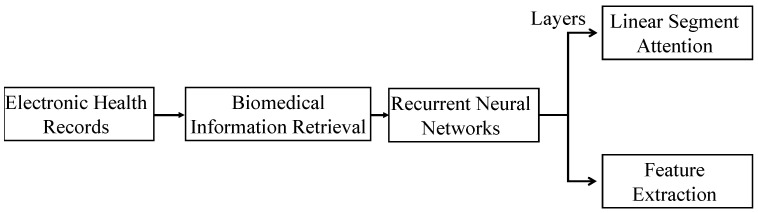
EHR framework for enhancing healthcare through EHRs and BIRs.

The proposed framework aims to provide readers with a comprehensive understanding of the introduced approach for enhancing healthcare through electronic health records (EHRs) [58,59] and biomedical information retrieval systems (BIRs), as shown in Figure 1. This framework outlines the pivotal role of EHRs in improving patient care by facilitating quick access to vital health information and fostering better communication among healthcare providers while reducing medical errors. It details the essential components of an EHR system, including a patient data database, user interface, and various applications that empower clinicians with tools such as electronic prescribing and clinical decision support. In the context of BIRs, the framework emphasizes the significance of these software solutions in handling the vast and constantly expanding biomedical literature [60]. It highlights the techniques employed, such as natural language processing, machine learning, and artificial intelligence, for indexing and retrieving valuable information from this extensive source. The main challenge addressed is the management of the overwhelming volume of data in biomedical research. Furthermore, the manuscript introduces the application of recurrent neural networks (RNNs) in clinical studies, particularly in the recognition of named entities [61]. It focuses on the use of supervised learning LSTM models to construct an unstructured information (UI)–based clinical management approach, enabling the retrieval of information related to biomedical entities. Additionally, feedforward networks (FFNs) and NLP-based features are integrated to enhance clinical named entity recognition (cNER) methods. The proposed framework thus offers readers a clear and structured understanding of the approach’s components and its potential to revolutionize healthcare and biomedical information retrieval.

### 3.1. Electronic Health Records Architecture

Electronic health records (EHRs) are digital forms of a patient’s paper chart. They contain all of the patient’s health information, including demographics, medical history, medications, allergies, immunizations, and test results. EHRs can be used to improve the quality of care by providing clinicians with access to patient information at the point of care, improving communication among healthcare providers, and reducing medical errors [58,59]. There are a number of different EHR architectures, but they all share some common features. An EHR system typically consists of a database, a user interface, and a set of applications. The database stores the patient’s health information. The user interface allows clinicians to access and enter patient information. The applications provide clinicians with tools to use the patient’s health information, such as electronic prescribing, order entry, and clinical decision support. The EHR architecture is designed to meet the needs of the organization that will be using it. However, the factors to consider include the size of the organization, the number of clinicians who will be using the system, and the types of applications that will be used.

In addition, for electronic records of patient healthcare, we used pretrained word vectors learned on PubMed articles with word-to-vector (w2v) [62,63,64]. Since there is no evidence to suggest that CBOW outperforms skip-gram architecture for w2v, we arbitrarily selected skip-gram architecture. Combining n-gram textual features with rich behavior features can improve node prior computation performance. Despite this, textual and nontextual features are typically represented differently, and they are not linearly correlated.

### 3.2. Biomedical Information Retrieval (BIR) Approach

A biomedical information retrieval system (BIR) is a software system that enables users to find information stored in biomedical literature. BIRs use a variety of techniques to index and retrieve information from biomedical literature, including natural language processing (NLP), machine learning (ML), and artificial intelligence (AI) [17,65]. One of the most important challenges in developing a BIR is the sheer volume of data that need to be indexed. Biomedical literature is vast and ever growing, and it can be difficult to keep up with the latest research. Additionally, biomedical literature is often written in a technical jargon that can be difficult for nonexperts to understand. Another challenge is the need to protect patient privacy. BIRs must be designed to protect patient privacy by preventing unauthorized access to patient data. This can be done by using a variety of security measures, such as encryption and access control. Despite the challenges, BIRs have the potential to improve the efficiency and effectiveness of biomedical research by making it easier for researchers to find the information they need. BIRs can also be used to improve patient care by providing patients with access to the latest research on their condition.

**Figure 2:**
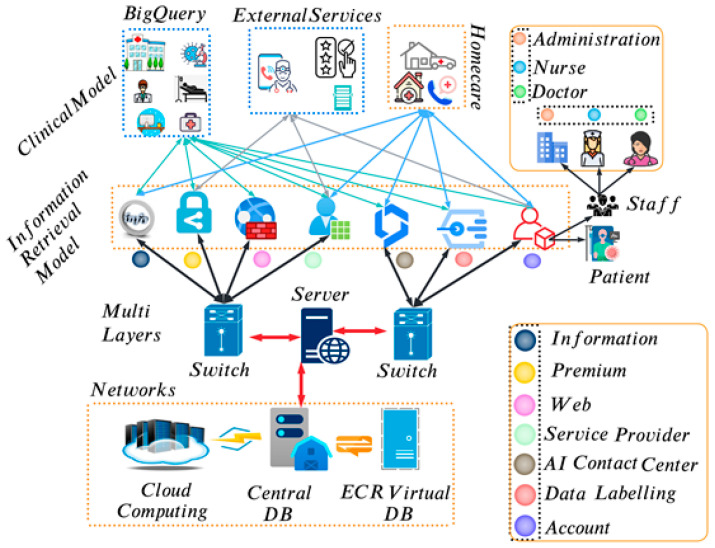
EHR architecture pipeline starting from the querying and indexing.

MEDREADFAST is a biomedical information retrieval system that was developed by researchers at the University of Pittsburgh [66]. It is designed to help clinicians find information quickly and easily in electronic health records (EHRs). MEDREADFAST uses a variety of techniques to index and retrieve information from EHRs, including natural language processing (NLP), machine learning (ML), and artificial intelligence (AI). MEDREADFAST has been shown to be effective in helping clinicians find information in EHRs [60]. In one study, MEDREADFAST was able to help clinicians find relevant information in EHRs 2.5 times faster than they could without MEDREADFAST. MEDREADFAST has also been shown to be effective in improving the quality of care that clinicians provide. In another study, MEDREADFAST was able to help clinicians identify and diagnose patients with pneumonia more accurately than they could without MEDREADFAST. MEDREADFAST is a valuable tool for clinicians. It can help them find information quickly and easily in EHRs, which can save time and improve the quality of care that they provide. MEDREADFAST is freely available for research use. Models are created using latent semantic indexing (LSI) algorithms and datasets obtained from the Health Improvement Network (HIN). LSI is an NLP technique that enables rich search results without revealing hidden relationships between terms, such as terms that are closely related. Since LSI mathematical models are complex and require large amounts of memory, this technique is not scalable, as shown in Figure 2.

Documents are classified as relevant or irrelevant based on their relevance to the user’s information requirements as a search query in an IR problem. Data from large collections, such as EHRs, must be collected in this manner to be relevant. To identify relevant cases and conduct correlational studies, translational research collects detailed clinical information, including disease stage [67], entry of the patient, severity, type of disease, recommended doctor, doctor’s observations, and patient response to cancer treatment. Search engine indexes are created by combining text characteristics and conceptual codes, thus allowing users to easily access documents.

### 3.3. Recurrent Neural Networks (RNNs)

**Figure 3:**
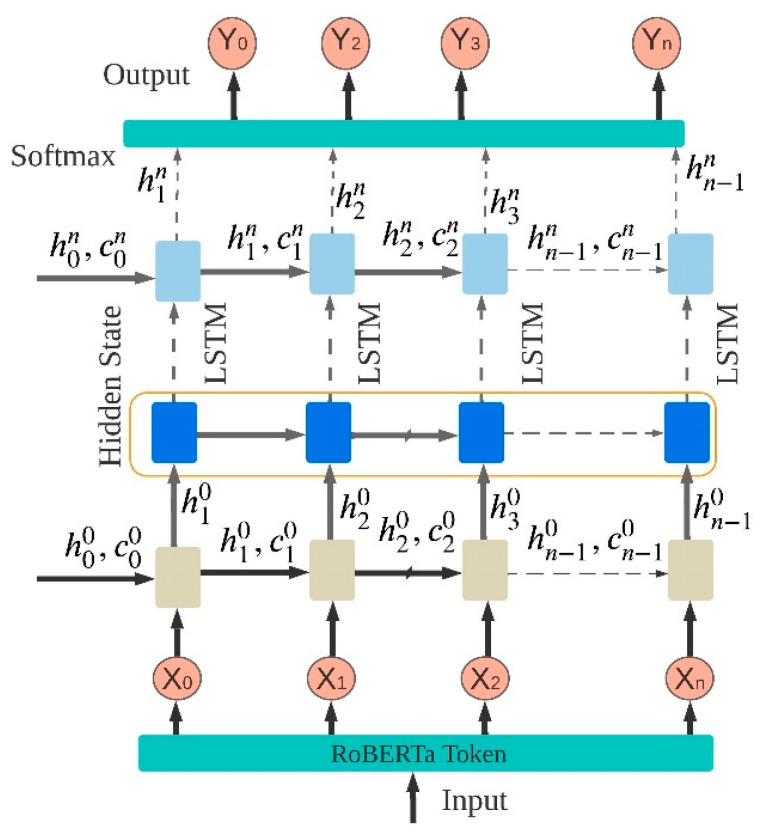
RNN framework for input, hidden layer, and output in BIR system.

RNNs on BIR are described in this subsection, which explains how they are used in different clinical studies. RNNs have opened up new avenues of research in sequence labeling [68]. Therefore, RNNs have been hard to train through backpropagation, because learning long-term dependencies using simple recurrent neurons lead to problems like report or fading gradients. An RNN-based A supervised model that uses terminology has been shown to improve recognition results [61]. In order to identify the named entities, we created an annotated corpus. In this study, a supervised learning LSTM is used to construct an unstructured information (UI)–based clinical management approach, as shown in Figure 3. A final step involved the use of the RNN hybrid system to retrieve information about biomedical entities (drugs, disease symptoms, therapeutic rules, etc.) that had been tokenized prior to being sent into a hidden state. Feedforward networks (FFNs) were used in the development of a clinical NER method (cNER) [69]. Feature extraction was performed using a w2v model, which exploited NLP-based features in the preprocessing stage. These methods can improve results by improving data quality and clinical task complexity.

#### 3.3.1. EHR Feature Extraction Layer

In this section, we provide a summary of the information extraction for cancer-related cancer treatment from EHRs that is successfully performed. Deep learning methods have succeeded great achievement in many domains through deep hierarchical feature construction and capturing long-range dependencies in data in an effective manner [70]. However, these methods have needed a huge extent of manual feature engineering and ontology mapping, which is one reason why such methods have seen limited adoption. Therefore, a predefined entity or relationship of interest is selected to be used as a feature of interest by IR to extract medication-related information [46]. In addition, such features include the hospital category, health condition, type of medication, dosage, entry, mode, bladder, frequency, and doctor information, as shown in Table 3; hospital treatment summaries include other information, such as hospital category, disease, medication names, medication types, dosages, entry, mode, reason, and symptom information. For example, free-text medical records would have to be converted into structured records with predefined slots and fillers filled with relevant data.

The best treatment for breast cancer depends on the stage of the cancer, the patient’s overall health, and the patient’s preferences. However, treatment may include surgery, chemotherapy, radiation therapy, hormone therapy, or targeted therapy. Early-stage cervical cancer can often be treated with surgery or radiation therapy. More advanced cervical cancer may require a combination of surgery, radiation therapy, and chemotherapy. Treatment may include surgery, chemotherapy, radiation therapy, immunotherapy, or a combination of these treatments.

In addition, targeted therapy uses high-energy rays to kill cancer cells [71]. However, radiation therapy can be given externally (from a machine outside the body) or internally (by placing radioactive material inside the body). Therefore, radiation therapy is used to shrink tumors, kill cancer cells that have spread to other parts of the body, or prevent cancer from coming back after treatment. Moreover, chemotherapy is the use of drugs to kill cancer cells. Chemotherapy drugs can be given by mouth, by injection, or through a vein. Therefore, raloxifene is a medication that is used to prevent and treat osteoporosis in postmenopausal women and those on glucocorticoids. It is also used to reduce the risk of breast cancer in those at high risk. Furthermore, raloxifene is a selective estrogen receptor modulator (SERM), which means that it acts like estrogen in some tissues, but not others.

Moreover, cervical cancer is treated with teletherapy, brachytherapy, and radiation therapy. Teletherapy uses a linear accelerator to deliver radiation from a distance. However, brachytherapy uses a radioactive source to deliver radiation from within the body. Moreover, radiation therapy is used alone or in combination with other treatments, such as surgery or chemotherapy. However, the targeted therapy is a type of treatment that uses drugs to target specific molecules on cancer cells. Furthermore, immunotherapy is a type of treatment that uses the body’s own immune system to fight cancer.

#### 3.3.2. EHR Linear Segment Attention Layer

**Figure 4:**
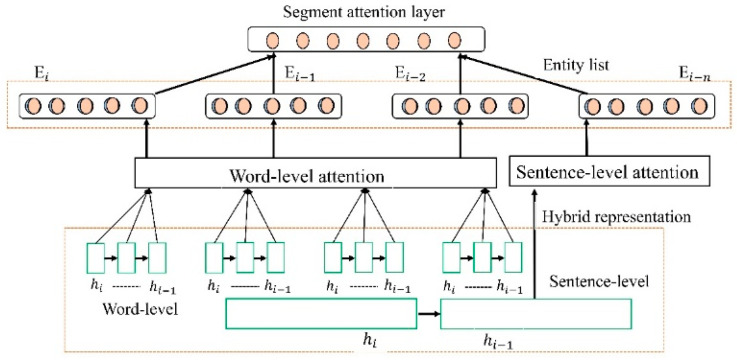
Electronic health record linear segment attention layer pipeline.

The RNN models sequential data using feedforward neural networks. The hidden state of neural networks is updated as each time step is received so that they can predict the outcome based on the inputs they receive. Since RNNs have a recurrent structure, they are capable of processing sequence data. With this model, the hidden unit will be updated at each time step, and the length of the sequences (sentences) will not be restricted. The fixed sentence length achieves the best results in available data [71], with I2B2 datasets. Variable sentence length is more difficult to represent sentence semantic information, and the interaction between sentences and entities far from retrieving information becomes weaker. The extraction, context text, and entity feature achieved the best results in the 2010 challenge reported, which enhanced performance [16]. However, both methods are not considered features in the current DL model. This study proposes a linear segmentation attention layer to overcome these limitations. This study proposes a linear segmentation attention layer to overcome these limitations, as shown in Figure 4.

LSTM used networks for predicting diagnoses (1–128), using target replication at each time step along with supporting targets for less-common diagnostic labels as a form of regularization [72]. In addition to LSTMs and bidirectional LSTMs, gated recurrent neural GRU tensor networks (GRN-TNs) enable the model to handle various types of sequence data [73,74]. BiRNN [75,76], is an early RNN model where forward and backward computations are carried out by the neurons. As a result of high-dimensional hidden states and nonlinear evolution, RNNs provide accurate predictions throughout many steps. Iterating over time creates an extremely rich dynamic because each unit uses simple nonlinearity. To compute hidden input states ($h_{Input}$ = $h_{1}$, $h_{2}$,…. $h_{n}$,) in a input sequence ($S_{Input}\;$= $x_{1}$, $x_{2}$,…. $x_{n}$), input vectors and output states (y_n_ = $y_{1}$, $y_{2}$,…. $y_{n}$). This creates text based on equations from 1 to *n*, as shown in Equations (1)–(5).

(1) $$h_{t}=\tanh(W_{hx}x_{n}+W_{hh}h_{t-1}+b_{h})$$

(2) $$h_{t}=\tanh(W_{input}+W_{hidden}+b_{h})$$

$$W_{input}=W_{hx}x_{n},\;$$

$$W_{hidden}=W_{hh}h_{t-1}$$

$$O_{t}=W_{oh}h_{t}+b_{o}$$

(3) $$O_{t}=W_{oh}\left(\tanh(W_{hx}x_{n}+W_{hh}h_{t-1}+b_{h})\right)+b_{o}$$

(4) $$O_{t}=W_{oh}\tanh(W_{hx}x_{n}+{W_{oh}W}_{hh}h_{t-1}+W_{oh}b_{h})+b_{o}$$

(5) $$O_{t}=\frac{1}{{\tanh^{-1}(W}_{oh}W_{hx}x_{n}+{W_{oh}W}_{hh}h_{t-1}+W_{oh}b_{h})+b_{o}^{-1}}$$

A weight matrix is an expression that indicates whether a feature vector for input ($i_{h}$), output $o_{h}$ hidden state, and hidden-to-hidden ($h_{h}$) are signified with $W_{xh}$, $W_{hh}$, and $W_{oh}$, respectively.

### 3.4. Evaluation Metrics

Evaluation metrics are used to measure the performance of a machine learning model, thus allowing us to quantify how well our models are able to make accurate predictions on unseen data. They are used to compare different models and to track the performance of a model over time. There are many different evaluation metrics available, each of which is suited for a particular type of model or task. We utilized the accuracy and F1-score as a primary metric, while P and R are secondary metrics in our case, as in Equation (6). The F1-score strikes a balance between recall and precision, making it a valuable metric when we need to consider both aspects of classification performance. In contrast, accuracy can be a reliable measure primarily when class distribution is balanced, as it equally weighs correct predictions across all classes:

(6) $$F_{1}=\left(\frac{Precision\times Recall}{Precision+Recall}\right)\times 2$$

where TP is true positive, and FP and FN is false positive and false negative, respectively. Performance metrics quantify the performance of models. The following classification metrics were used to assess the model’s overall viability as a classifier and its performance. The accuracy prediction is a model in the context of classification, which is the ratio of correct predictions over the total number of examined instances, as in Equation (7).

(7) $$\mathrm{accuracy}=\frac{TP+TN}{TP+TN+FP+FN}.$$

Precision measures positive patterns that are correctly predicted over the total positive prediction patterns, as in Equation (8). Additionally, the precision is written as macro and weight average precision, as in Equations (9) and (10).

(8) $$\mathrm{Precision}=\frac{TP}{TP+FP}$$

(9) $$\mathrm{Macro}\;\mathrm{average}\;\mathrm{precision}=\frac{\sum_{i=1}^{l}\frac{{TP}_{i}}{{TP}_{i}+{FP}_{i}}}{l}$$

(10) $$\mathrm{Weight}\;\mathrm{average}\;\mathrm{precision}=\frac{\sum_{i=1}^{l}\frac{{TP}_{i}}{{TP}_{i}+{FP}_{i}}\;\;X\;n_{i}}{l}$$

Recall is a measure of positive patterns over the total correct predictions. Recall is calculated for each class; thus, averaging is essential for multiclass model calculation, as in Equations (11)–(13).

(11) $$\mathrm{Recall}=\frac{TP}{TP+FN}$$

(12) $$\mathrm{Macro}\;\mathrm{average}\;\mathrm{precision}=\frac{\sum_{i=1}^{l}\frac{{TP}_{i}}{{TP}_{i}+{TN}_{i}}}{l}$$

(13) $$\mathrm{Weight}\;\mathrm{average}\;\mathrm{precision}=\frac{\sum_{i=1}^{l}\frac{{TP}_{i}}{{TP}_{i}+{TN}_{i}}\;\;X\;n_{i}}{l}$$

## 4. Implementation and Results

As part of this section, we first describe the experimental setup and baselines, followed by an analysis of the empirical results and a comparison of various models with varying features.

### 4.1. Data Preprocess

**Figure 5:**
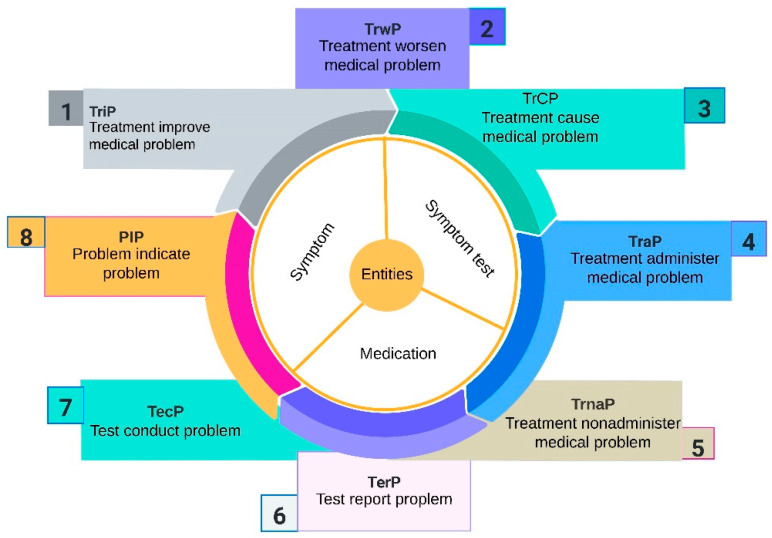
Overall EHR summary of three types of entities, including symptom, test, and treatment, and eight types of relations to entities.

**Figure 6:**
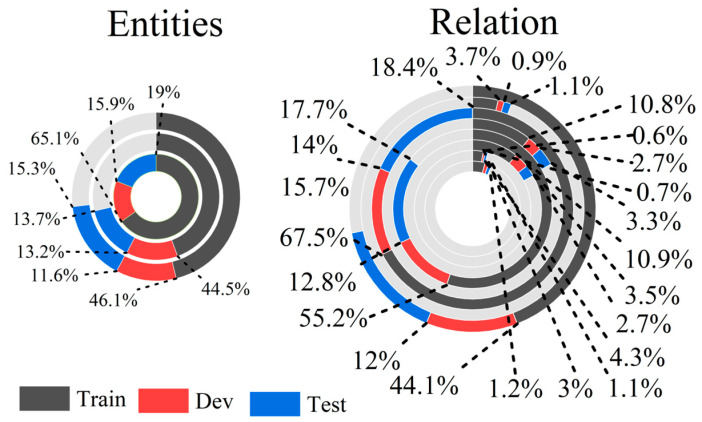
Types and statistics of the I2b2 dataset. The light grey color represents the statistics of TrAP, TrIP, TrCP, TrWP, TrNAP, PIP, TeCP, and TeRP in each circle. For example, TrAP represent Train, Dev, and Test data in the single circle, the statistics of TrAP is compared to the rest of data in the same circle.

We used a I2B2-2010 shared task challenge dataset [77]. Three types of entities and eight types of relationships were manually annotated by experts on discharge summaries from three different hospitals. Types of entities included symptom, test, and treatment/diagnosis of cancer. While the types of relations including treatment are administered for medical problem (TrAP), treatment improves medical problem (TrIP), treatment causes medical problem (TrCP), treatment worsens medical problem (TrWP), treatment is not administered because of medical problem (TrNAP), medical problem indicates medical problem (PIP), test is conducted to investigate medical problem (TeCP), and test reveals medical problem (TeRP). Training, development, and test sets were split at a 60:20:20 ratio at random. Thus, combining each baseline and feature, besides that from the datasets of balance data distribution (BDD), we also made comparisons on nonbalance distribution (NDD) data. The data preprocessing process consists of cleansing data and removing data noise based on the adopted strategy. Therefore, EHR data should be processed according to reasonable methods, especially for the preprocessing of the data, as shown in Figure 5. The statistics of this dataset is shown in Figure 6.

### 4.2. Parameter Tuning

In our model, we initialized parameters with pretrained 50-dimensional word embeddings. In addition, we tuned the parameters on the validation set by random search. The primary parameters of our model were fitted with the same values, as shown in Table 4. The number of epochs was chosen by an early stopping strategy on the validation set [78]. We used the five different scenarios (cases 1–5) to configure multiple parameters, which helped to analyze the same task and compare the evaluation performance.

#### 4.2.1. Baseline Discussion

**BioBERT** is a BERT model that has been pretrained on a biomedical corpus [54]. It is specifically designed for biomedical natural language processing tasks, such as named entity recognition and relation extraction.

**ClinicalBERT** is a BERT model that has been pretrained on a clinical corpus [55]. It is specifically designed for clinical natural language processing tasks, such as question answering and clinical decision support.

**BioBERT-uncase** (https://huggingface.co/cambridgeltl/BioRedditBERT-uncased, accessed on 28 June 2023) is a version of BioBERT that has been trained on a corpus of text that has been case-insensitively tokenized [79]. This makes it more efficient for tasks that do not require case-sensitive tokenization, such as text classification.

**RoBERTa-case** (https://huggingface.co/Finnish-NLP/roberta-large-finnish-v2, accessed on 28 June 2023) is a version of RoBERTa that has been trained on a corpus of text that has been case-sensitively tokenized. This makes it more accurate for tasks that require case-sensitive tokenization, such as named entity recognition.

These models are all based on the Transformer architecture, which is a neural network architecture that has been shown to be very effective for natural language processing tasks. They have all been pretrained on large corpora of text, which allows them to learn the statistical relationships between words and phrases. This makes them very accurate at a variety of natural language processing tasks, such as text classification, relation extraction, and question answering.

#### 4.2.2. Results and Discussion

We outlined n-gram and rich features using the empirical results of I2b2 datasets listed in Table 5 and Table 6. We performed five random runs for each kind of test data and reported the average results. We also noted that the BioBERT and RoBERTa models fine-tune with the attention-based method, initially, for baseline. In addition, we used these models without fine-tuning (baseline1 and baseline2). Therefore, the RoBERTa-base and RoBERTa-large models were used to study how they perform on biomedical tasks. After being pretrained with larger batch sizes than BERT, both strategies used dynamic masking strategies to prevent overmemorization. The I2B2 datasets weighted BDD, outperformed using NBD. Furthermore, the performance of BDD was also significantly better than that of NDD across all models and features, as can be seen in Table 5 and Table 6. Since the precision of NDD was below the precision of BDD, retrieving information in an imbalanced class distribution was much more difficult.

Moreover, the recall of the proposed model on NDD in Table 5 and Table 6 implies that nearly half of the information in the test dataset is accurate. Prediction models are often poor when there is high data imbalance [80]. However, the BDD on I2b2 yielded a higher evaluation score, including F1-score, precision, and recall, with an F1-score of 84.66%, a precision of 85.54%, and a recall of 83.80%. Meanwhile, the average macro F1-score was 64.63%, precision was 65.51%, recall was 63.77%, and accuracy was 88.4% on test set.

Meanwhile, for the validation set, the weighted averaged F1-score was 85.08%, precision was 85.93%, and recall was 84.25%, and the average macro F1-score was 65.47%, precision was 66.89%, recall was 54.43%, and accuracy was 89%. Based on the same parameter tuning, we propose a model for testing data.

The evaluation results on EHR datasets are obtained using hyperparameter tunning cases 1–5. In terms of the evaluation performance of case 1 on baseline 1, 27% accuracy was achieved; case 2, 29% accuracy; BioBERT-CRF, 47% accuracy; RoBERTa-LSTM, 49% accuracy; and BioBERT-CRF, 51% accuracy; meanwhile, our proposed model utilized EHR data and achieved 59% accuracy. Case 2 performed well on baseline2 in terms of evaluation accuracy, with 41% accuracy; RoBERTa-CRF, 50% accuracy; BioBERT-CRF, 48% accuracy; RoBERTa-LSTM, 54% accuracy; and BioBERT-CRF, 58% accuracy, and our proposed model on case 3 performed well in terms of accuracy, with 64% accuracy. According to our evaluation performance of case 3 on baseline 1, RoBERTa-CRF achieved 63% accuracy, BioBERT-CRF achieved 51% accuracy, RoBERTa-LSTM achieved 68% accuracy, BioBERT-CRF achieved 66% accuracy, and our proposed model on case 3 achieved 76% accuracy with EHR data. A higher evaluation performance was achieved in case 4, while in RoBERTa-CRF, we were able to achieve 76% accuracy, which was outperformed compared with our proposed model and the BioBERT-CRF model. Moreover, BioBERT-CRF achieved an evaluation performance of 74%. According to our proposed model on case 4 on EHR data, the accuracy was 68%.

In addition, case 5 was evaluated better than cases 1–4, in which baseline 1 achieved 54% accuracy. However, baseline 2 achieve 66% accuracy; F1-score, 0.614; precision, 0.632; and recall, 0.597. Baseline 2, which uses BioBERT-uncased, achieved lower results than other models in some tasks, such as RoBERTa and BioBERT. However, BioBERT-uncased still achieved good results on other tasks, such as question answering (QA). This suggests that baseline 2 is a promising model for biomedical text mining, and it could be further improved by training it on a larger dataset and fine-tuning it on specific tasks. Moreover, RoBERTa-CRF achieved 76% accuracy; F1-score, 0.730; precision, 0.725; and recall, 0.735. The RoBERTa-CRF model is trained end to end, which means that the parameters of both RoBERTa and CRF are learned jointly. This allows the model to learn the long-range dependencies between words that are present in the RoBERTa embeddings, as well as the short-range dependencies between labels that are present in the CRF model. BioBERT-CRF achieved 57% accuracy; F1-score, 0.537; precision, 0.530; and recall, 0.544. It has been shown to be more accurate than other NER models, such as BiLSTM-CRF and CRF-based models. BioBERT-CRF is also more robust to noise and can handle out-of-domain data better than other NER models. RoBERTa-LSTM achieved 81% accuracy; F1-score, 0.757; precision, 0.745; and recall 0.769. BioBERT-CRF achieved 86% accuracy; F1-score, 0.806; precision, 0.815, and recall, 0.797.

Furthermore, this study proposed a model on case 5 that shows higher evaluation performance using EHR data with an accuracy of 89%, F1-score of 0.8466, precision of 0.8559, and recall of 0.8375 on our proposed models. As you can see, the newer proposed models outperform the BERT-BiLSTM-CRF, BioBERT-CRF, and RoBERTa-CRF models. This is likely due to the fact that the newer models were trained on larger and more diverse datasets. Additionally, the newer models were fine-tuned on the specific task of named entity recognition, which helped to improve their performance. We were able to accomplish this work much more easily with the help of many existing NLP tools and knowledge resources, allowing us to use this approach to extract relations. In clinical texts, these methods demonstrate that deep learning is effective for relation extraction. Our model evaluation performance using case 5 is compared with that of Sahu et al. [81], Rink et al. [16], Patrick et al. [77], Divita et al. [82], Bhatia et al. [83], and Ji et al. [84] on an I2B2 dataset, as shown in Table 7.

**Figure 7:**
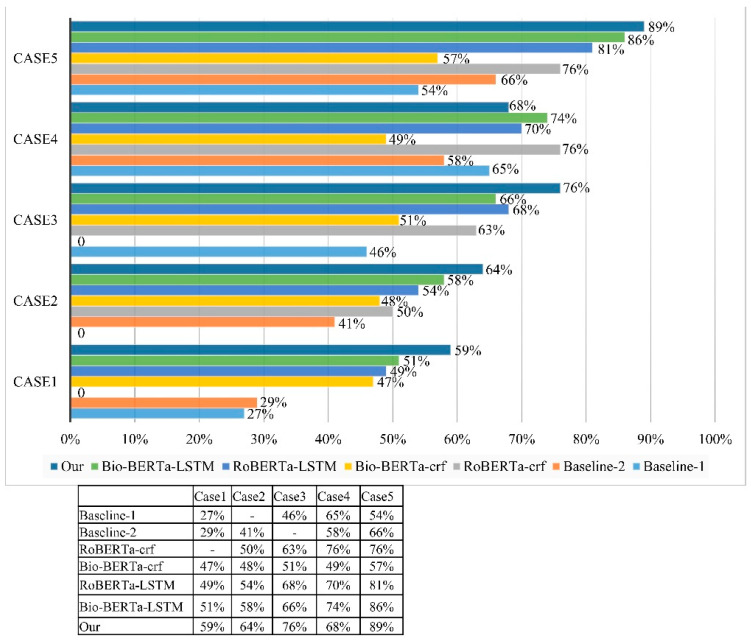
An evaluation performance metric boxplot is presented to illustrate algorithm accuracy.

Comparing the accuracies, our model performed better in the TrWP, Medic, and TrCP relations in the information retrieval system. As a result of the lack of enough instances and additional preprocessing, our model’s score on TeRP, TeCP, PIP, and TrAP decreased slightly. TeCP was achieved by Bharatia [83] at 82%; PIP on Divita [82], 71%; Medic in our model, 79%; and TrAP, 71.6% on a Sahu model [16]. Fortunately, our model greatly improved over the above-proposed models in retrieving the TrWP, Medic, and TrCP relations on I2B2 data. Medical informatics researchers can focus on new problems using high-quality, freely available NLP tools for extraction, retrieval, and data mining. Information extraction on IRD datasets on five cases of hyperparameters was performed, as shown in Figure 7.

Our proposed model results showed that each set of features advances the extraction of all case relations. However, some individual features provide information that is more useful to the extraction of a specific relation. In BIR and translational research, this is one of the most important and fundamental tasks to be addressed using DL models, which have been successful in tackling this task. In terms of the evaluation performance of case 1, low accuracy was achieved on baseline 1 and baseline 2, at 27%, and 29%, while it achieved higher in case 5, 54% and 66%, respectively. We also observed that worse results were achieved in RoBERTa-CRF case 1 ‘0’, as well as in case 2 and case 3 on baseline 1 and 2, respectively. According to our proposed model in case 4 and case 5, the accuracy was 68% and 89%, respectively.

### 4.3. Implications

#### 4.3.1. Theoretical Implications

This study’s findings make the following theoretical implications: First, it contributes to the existing knowledge by demonstrating the critical role of the framework presented, including a shift towards integrating advanced technologies, such as machine learning and artificial intelligence, into healthcare and biomedical research [17,65], investigating and emphasizing the need for tailored electronic health record (EHR) architectures that consider factors like organization size and application requirements [1]. The challenges of biomedical information retrieval underscore the importance of developing more sophisticated indexing and retrieval methods [58]. Therefore, this study’s findings contribute to our understanding of novel techniques for retrieving biomedical information of related or similar EHR between medical problems during symptom detection in existing information through testing and predicting the treatment in hospital clinical procedures.

Second, this study utilized Transformers, which play a crucial role in BIR for cancer treatment within EHR. Their exceptional, contextual understanding, sequence-to-sequence capabilities, and efficiency in handling large-scale data are invaluable in processing complex medical records [62,63,64]. As a result, Transformers can seamlessly integrate diverse data types, including text, images, and structured data, enhancing their utility in aggregating patient information. This study used pretrained models like BERT [51] and GPT, thus providing a substantial knowledge base for BIR. Their interpretability and adaptability improve the understanding of treatment recommendations and enable continuous updates with the latest medical knowledge. Transformers’ swift data retrieval from EHRs aids in timely decision making in cancer treatment, ultimately enhancing the quality of patient care in healthcare [59]. This study could lead to significant improvements in the efficiency and accuracy of clinical trials, as well as the development of new insights into disease progression and treatment response.

Finally, considering data distribution, especially in healthcare with imbalanced data, is critical when developing models for healthcare-related tasks. These theoretical implications suggest a growing reliance on technology, customization, and data management in healthcare and biomedical fields [17]. As healthcare data become increasingly complex and voluminous, it will be essential to develop new and innovative ways to collect, manage, and analyze these data. This study will require a close collaboration between healthcare professionals, data scientists, and engineers for further insight.

#### 4.3.2. Practical Implications

This study provides substantive practical implications for healthcare organizers to include reducing redundant tests, enhancing care coordination, and providing timely and accurate information to healthcare professionals. First, the importance of attitude and subjective norms in shaping the focus on electronic health records (EHR) and biomedical information retrieval (BIR) carries significant implications for the healthcare industry. It underscores the potential for enhancing patient care through improved information accessibility, reduced medical errors, and enhanced communication among healthcare professionals. Successful EHR and BIR implementation hinges on the willingness and support of both healthcare providers and organizations. Understanding the factors influencing these attitudes and norms can guide strategies to encourage technology adoption. This can be achieved through training and education for providers to grasp the benefits and effective use of these technologies, as well as by introducing incentives, such as government financial support, to drive their adoption. 

Second, this research reveals the critical nature of software tools: Mention of tools like MEDREADFAST, which is designed to help clinicians access information in EHRs quickly, highlights the practical use of software solutions in healthcare. Such tools can significantly improve the efficiency of healthcare professionals and the quality of care they provide. A healthcare organization is using this study to track the quality of care that is being provided. The tool collects data from a variety of sources, including patient surveys and clinical outcomes data.

Third, the effectiveness of the discussion of data preprocessing methods emphasizes the importance of preparing healthcare data for analysis. This finding has practical implications for data quality and the successful implementation of machine learning models in healthcare.

## 5. Conclusions and Future Work

This study presents a multifaceted approach to address the challenges and opportunities in biomedical information retrieval (BIR) within electronic health records (EHRs). With the expanding landscape of electronic health data, there is a growing need for innovative solutions to provide clinicians and researchers with swift access to critical patient information. Therefore, we introduced an enhanced biomedical language model empowered by biomedical knowledge graphs to elevate BIR tasks. A key contribution lies in our novel techniques for biomedical information retrieval within EHRs, focusing on symptom detection and treatment prediction. Our approach outperformed state-of-the-art BIR systems, excelling in learning semantic relationships within biomedical documents, even in the face of complex clinical texts.

Moreover, these study results lead to a broad outline of the strengths and weaknesses of diverse models in terms of predictive performance and training efficiency. First, in terms of performance, we meticulously examined the performance of different models across a spectrum of evaluation criteria, focusing on predictive accuracy and training/decoding efficiency. Specifically, we modified a standard linear chain, through rigorous testing on the I2b2 datasets; we conducted five random runs for each test data category and reported average results. Our evaluation encompassed various strategies, including fine-tuning BioBERT and RoBERTa models with attention-based methods as baselines. We observed that the weighted balance data distribution (BDD) outperformed the nonbalance distributed data (NDD) in terms of precision, particularly highlighting the challenges of retrieving information in imbalanced class distributions.

Interestingly, the accuracy levels achieved via these methods are higher than the intercoder agreement levels, as evaluated on the same test data and according to the same evaluation of accuracy/agreement. This evaluation is especially noteworthy since the feature set used in our work is fairly standard, as widely believed, as remarked in [3,4]. Our results, obtained via a BDD system, achieved a remarkable precision, recall, and F1-score of 85.54%, 83.80%, and 84.66%, respectively, underscoring its superiority. In particular, the experiments we conducted demonstrated robust performance across different evaluation cases on EHRs datasets, achieving notable accuracy and outperforming several baseline models. It is worth noting that the RoBERTa-CRF and BioBERT-CRF models showed promising results but with increased complexity. Overall, our findings shed light on the strengths and limitations of various models, offering valuable insights into their practical utility in biomedical information extraction tasks.

In essence, our proposed approach stands as a testament to the transformative potential of cutting-edge technology in the realm of healthcare informatics, offering a pathway towards more informed, efficient, and effective healthcare delivery. We anticipate that our research will inspire further exploration, collaboration, and advancements in this critical field, ultimately benefiting both clinicians and patients alike.

## Figures and Tables

**Table 1:** EHR sample list with real-time example.

	Clinical Text Problem Samples
$S_{1}$	Doctor: “He was given Lexix to prevent him from congestive heart failure.”
$S_{2}$	People who are currently diagnosed with cancer, including breast cancer, have a higher risk of severe illness if they get COVID-19.
$S_{3}$	Chemotherapy and immunotherapy can weaken the immune system and possibly cause lung problems.
$S_{4}$	Pneumonia is an infection that inflames the air sacs in one or both lungs.

**Table 2:** Different past biomedical information extraction techniques and limitations.

Information Extraction Techniques	Proposed Method	Limitation
Biomedical information in EHR [42]	Combination of multimodel techniques and tools	A current user interface and the usefulness of the search features
Document’s text to a keyword style query [43]	Query-document delta matrix passed through deep feedforward	A relatively small amount of training data
Bayesian learning approach [44]	Biomedical IR performance through diversity and a reranking algorithm	TREC 2004–2007 Genomics datasets
Biomedical domain knowledge IR [45]	A cross-linguistic framework for monolingual and concept-based retrieval of biomedical information	Concept-based retrieval and user system communication
Biomedical query expansion [46]	Pseudo-relevance feedback method based on mesh, which combines information with a corpus	Extracting biomedical feature resources for optimizing expansion term refinement
Learning manual information [47]	Optimal ranking strategy and groupwise learning boost the diversity of retrieved relevant documents	Automatic aspect mining when the dataset contains no such annotations
Tool for Electronic Medical Record Search Engine (EMERSE) [48]	EMERSE is a Web-based application that supports cancer research online (http://www.webmd.com/cancer/ and http://www.cancer.gov/, accessed on 28 March 2023)	Involves securely networking sites for obfuscated counts
Point of healthcare IE [49]	Clinical care or healthcare IR systems	Manual healthcare IR
Electronic medical record [50]	Primarily investigated triresearch questions medical IR	Inclusion of entity attributes, web text preprocessing, and cross-validation

**Table 3:** Feature extraction for patients ($P_{i})\;$*i* = 6 with diverse syndromes and cancer treatment method.

Patient Feature	$P_{1}$	$P_{2}$	$P_{3}$	$P_{4}$	$P_{5}$	$P_{6}$
Record	101	102	103	104	105	106
Syndrome	Breast neoplasm	Cervical neoplasm	Lung cancer	Breast neoplasm	Lung cancer	Breast neoplasm
Treatment	Hormone therapy	Teletherapy	Immunotherapy	Hormone therapy	Immunotherapy	Hormone therapy
	Chemotherapy	Brachytherapy	Targeted therapy	Chemotherapy	Targeted therapy	Chemotherapy
	SERMs	Radiation therapy	Chemotherapy	SERMs	Chemotherapy	SERMs
Doctor	Oncologist	Oncologist	Oncologist	Oncologist	Oncologist	Oncologist
Dosage	21–60 mg	0.40–2.0 Gy/h	58–73 Gy	31–51 mg	46–62 Gy	21–51 mg
Mode	nm	-	-	nm	-	nm
Frequency	q.d	-	-	q.d	-	q.d
Duration	6 months	55 days	4 months	2–3 months	3–6 months	3 months
Reason	Healthy	Healthy	Death	Healthy	Healthy	Death
Gender	F	F	F	F	M	F
Stage	I	II	I	II	III	I

**Table 4:** Parameters with their detailed values for five different scenarios.

Hyperparameters	Case 1	Case 2	Case 3	Case 4	Case 5
Learning rates	1 × $10^{-3}$	2 × $10^{-3}$	3 × $10^{-3}$	3 × $10^{-4}$	5 × $10^{-4}$
Epochs	30	20	20	10	15
Batch sizes	128	64	32	8	16
n_clusters	2	2	2	2	0
Dropout	0.4	0.4	0.2	0.2	0.3
Optimizer	Adamax	GD	RMSprop	Adamax	AdamW
Weight decay	0.1	0.01	0.01	0.1	0.1
Output layer	Softmax	-	-	Softmax	Softmax
Pretrain model	12	24	24	12	12
Kernel	1	1	1	1	3
Hidden Layers	768	768	768	768	768
Test size	0.6	0.5	0.4	0.3	0.2
Train size	0.4	0.5	0.6	0.7	0.8

**Table 5:** The evaluation metrics (macro and weight) of our model for parameter tuning case 5 on I2B2 data.

Distribution				Macro			Weight		
	Split	Instance	Acc	Prec	Rec	F1	Prec	Rec	F1
NBD	Test	20%	**78.4%**	0.5533	0.5371	0.5451	0.7655	0.7637	0.7646
	Valid	80%	**80%**	0.5683	0.5448	0.5563	0.7659	0.7641	0.7650
BDD	Test	20%	**88.4%**	0.6551	0.6377	0.6463	0.8554	0.8380	0.8466
	Valid	80%	**89%**	0.6689	0.5643	0.6547	0.8593	0.8425	0.8508

**Table 6:** The evaluation metrics of our model on a test set for parameter tuning cases 1–5.

Parameters	Metric	Baseline-1	Baseline-2	RoBERTa-crf	Bio-BERT-crf	RoBERTa-LSTM	Bio-BERT-LSTM	Our
Case 1	Acc	27%	29%	-	47%	49%	51%	**59%**
	F1	0.217	0.221	-	0.429	0.457	0.452	0.543
	P	0.203	0.236	-	0.435	0.443	0.468	0.530
	R	0.233	0.208	-	0.423	0.472	0.437	0.557
Case 2	Acc	-	41%	50%	48%	54%	58%	**64%**
	F1	-	0.337	0.445	0.425	0.498	0.519	0.614
	P	-	0.346	0.438	0.436	0.502	0.536	0.605
	R	-	0.328	0.452	0.415	0.494	0.503	0.623
Case 3	Acc	46%	-	63%	51%	68%	66%	**76%**
	F1	0.415	-	0.587	0.465	0.647	0.611	0.724
	P	0.405	-	0.560	0.458	0.651	0.625	0.713
	R	0.426	-	0.617	0.472	0.643	0.698	0.735
Case 4	Acc	65%	58%	**76%**	49%	70%	74%	68%
	F1	0.586	0.519	0.703	0.431	0.648	0.713	0.642
	P	0.600	0.537	0.715	0.445	0.652	0.704	0.655
	R	0.573	0.502	0.691	0.418	0.644	0.722	0.630
Case 5	Acc	54%	66%	76%	57%	81%	86%	**89%**
	F1	0.517	0.614	0.730	0.537	0.757	0.806	0.846
	P	0.494	0.632	0.725	0.530	0.745	0.815	0.855
	R	0.542	0.597	0.735	0.544	0.769	0.797	0.837

**Table 7:** The best accuracy of our model compared with the previous model on multiple data.

Models	TrCP	TeRP	TeCP	PIP	Medic	TrAP	TrWP
I2b2 2010							
Sahu et al. [81]	56.4%	11%	50.6%	64.9%	55%	71.6%	59%
Rink et al. [16]	55.4%	75%	51%	69.4%	76.4%	75.7%	64%
Patrick et al. [77]	48.7%	84%	50%	65.1%	-	71.2%	76%
Divita et al. [82]	48.5%	83.7%	37.7%	71%	55%	47.46%	68%
I2b2 2012							
Bhatia et al. [83]	17%	26%	82%	48%	56.3	-	78.9%
Ji et al. [84]	29.45%	55.95%	32.79%	21.67%	-	47.46%	48%
**Our**	**66%**	**87%**	57%	70%	69%	**81%**	**89%**

## Data Availability

Researchers interested in accessing the data for validation or further analysis can contact the corresponding author to discuss data availability and permissions.
